# Automatic and interpretable prediction of the site of origin in outflow tract ventricular arrhythmias: machine learning integrating electrocardiograms and clinical data

**DOI:** 10.3389/fcvm.2024.1353096

**Published:** 2024-03-20

**Authors:** Álvaro J. Bocanegra-Pérez, Gemma Piella, Rafael Sebastian, Guillermo Jimenez-Perez, Giulio Falasconi, Andrea Saglietto, David Soto-Iglesias, Antonio Berruezo, Diego Penela, Oscar Camara

**Affiliations:** ^1^ Physense, BCN Medtech, Department of Information and Communication Technologies, Universitat Pompeu Fabra, Barcelona, Spain; ^2^ Computational Multiscale Simulation Lab (CoMMLab), Department of Computer Science, Universitat de Valencia, Valencia, Spain; ^3^ Cardiology Department, Heart Institute, Teknon Medical Center, Barcelona, Spain; ^4^ Division of Cardiology, Department of Medical Sciences, University of Turin, Turin, Italy; ^5^ Department of Arrhythmology, Humanitas Research Hospital, Milan, Italy

**Keywords:** electrocardiogram, feature analysis, machine learning, outflow tract ventricular arrhythmias, site of origin

## Abstract

The treatment of outflow tract ventricular arrhythmias (OTVA) through radiofrequency ablation requires the precise identification of the site of origin (SOO). Pinpointing the SOO enhances the likelihood of a successful procedure, reducing intervention times and recurrence rates. Current clinical methods to identify the SOO are based on qualitative analysis of pre-operative electrocardiograms (ECG), heavily relying on physician’s expertise. Although computational models and machine learning (ML) approaches have been proposed to assist OTVA procedures, they either consume substantial time, lack interpretability or do not use clinical information. Here, we propose an alternative strategy for automatically predicting the ventricular origin of OTVA patients using ML. Our objective was to classify ventricular (left/right) origin in the outflow tracts (LVOT and RVOT, respectively), integrating ECG and clinical data from each patient. Extending beyond differentiating ventricle origin, we explored specific SOO characterization. Utilizing four databases, we also trained supervised learning models on the QRS complexes of the ECGs, clinical data, and their combinations. The best model achieved an accuracy of 89%, highlighting the significance of precordial leads V1-V4, especially in the R/S transition and initiation of the QRS complex in V2. Unsupervised analysis revealed that some origins tended to group closer than others, e.g., right coronary cusp (RCC) with a less sparse group than the aortic cusp origins, suggesting identifiable patterns for specific SOOs.

## 1 Introduction

Ventricular tachycardia (VT) is a serious heart rhythm disorder that can lead to sudden cardiac death (SCD). VT is responsible for an estimated 80% of SCD cases worldwide, highlighting the critical need for accurate diagnosis and treatment (1). Idiopathic ventricular arrhythmias (IVAs) pose a particular challenge in the VT spectrum, as the underlying mechanisms triggering these arrhythmias remain elusive. Among IVAs, outflow tract ventricular arrhythmias (OTVAs) are the most common type, originating from the outflow tracts of the ventricles, the regions connecting the ventricles to the major arteries. The structural and functional complexity of these structures makes diagnosis and planning treatment for OTVAs particularly difficult.

Current treatment options for OTVAs include antiarrhythmic drugs and radiofrequency ablation (RFA) (2). However, the effectiveness of RFA has been suboptimal, with excessively high recurrence rates reported (3). To improve the efficacy of RFA, accurate pre-operative planning is essential. This involves identifying the optimal ablation site, known as the ectopic foci or the site of origin (SOO) of the OTVA, prior the procedure.

The electrocardiogram (ECG) stands as a pivotal diagnostic instrument for identifying the SOO in OTVAs, where its morphological changes are discernible in affected patients (2). Initial estimates of the SOO, distinguishing between right (RVOT) and left (LVOT) ventricle origins, are often derived through visual inspection of the ECG.

Several methods for exploring ECG characteristics have been developed to identify LVOT and RVOT origins, which have been summarized in comprehensive reviews (4–6). For instance, some algorithms consider the proximity of the origin to specific ECG lead patterns, associating left bundle branch block (LBBB) patterns with origins near V1 and right bundle branch block (RBBB) patterns with origins further from the anterior chest. However, the intricacy arises as LVOT origins may exhibit either LBBB or RBBB patterns, contingent on the specific SOO. A critical element in these algorithms is the precordial transition (defined as the first precordial lead with a dominant R wave), which establishes criteria for determining the transition zone or analyzing the R/S wave ratio (the amplitude of the R wave divided by the amplitude of the S wave in the QRS complex) in specific leads. Despite their reported high accuracies, often surpassing 85%, many of these methods are tailored to data from a single center, they rely on visually estimated ECG characteristics and incorporate highly specific thresholds, limiting their generalizability. Other methods focus on analyzing the precordial transition of both sinus rhythm and premature ventricular contractions (PVCs), which are frequently present in OTVA cases (7, 8).

Nonetheless, the reliance on clinician expertise for visually interpreting these features introduces potential inconsistencies in the analysis. Moreover, precordial lead analysis is subject to variability based on electrode placement and heart rotation, complicating standardization of diagnostic criteria. Relying solely on visual ECG inspection may result in misdiagnosis and suboptimal intervention approaches, potentially prolonging procedural time and increasing recurrence risks.

Recently, advanced methods have been developed to enhance the accuracy of SOO prediction beyond visual inspection. These methods combine ECG visual morphology analysis with patient data to improve SOO classification. For instance, Penela et al. (9) proposed a hybrid algorithm that incorporates clinical features, such as sex, hypertension, and age, along with ECG analysis based on the precordial R/S transition and the amplitude in lead V3. However, this approach relies on manual thresholds for age and V3 amplitude, and it is partially dependent on clinician expertise in determining the R/S transition.

While these methods offer improvements over visual inspection alone, they still face limitations in terms of accuracy and interpretability. Doste et al. (10) proposed a machine learning (ML) approach using both real and simulated signals to classify OTVA origin. However, this approach does not consider patient-specific information, potentially limiting its generalizability to real-world clinical scenarios.

To address these limitations, our work proposes a comprehensive ML methodology that integrates signal analysis for both simulated and real data, patient-specific information, and a systematic exploration of the most relevant features. This integrated approach not only enhances classification accuracy but also provides valuable interpretability, offering clinicians insights into critical factors influencing treatment decisions. Furthermore, our proposal extends beyond classification to identify specific source sites through unsupervised algorithms, enabling a cluster analysis of different origins based on ECG similarities and patient characteristics. This holistic approach aims to significantly advance the precision and understanding of OTVA treatment planning.

## 2 Materials and methods

The study employed a comprehensive methodology to investigate the identification of OTVAs SOO, encompassing diverse databases and advanced analytical techniques. The main goal was to differentiate LVOT and RVOT origins with a supervised ML approach. Additionally, the specific SOO identification was explored with an unsupervised ML approach. Four distinct databases were utilized, comprising two with QRS complexes, one featuring simulated QRS complexes, and another incorporating QRS complexes along with clinical data from the patients.

In the supervised training phase, models were developed using QRS complexes and patient data independently. The training involved various combinations of QRS and patient data from the databases, exploring their impact on model performance. Additionally, an innovative approach was used, training models independently and using the inference from one as input for the other, with the goal of enhancing the predictive capabilities.

Furthermore, an algorithm was designed to automate the identification of the R/S transition from PVC and sinus rhythm beats, thereby improving the precision of the models. Additionally, all thresholds were removed from the original approach proposed by Penela et al. (9).

Feature relevance analysis was conducted using SHAP (SHapley Additive exPlanations) analysis (11) and Gini’s coefficient metric (12) on the QRS complexes models. This analysis informed the inclusion of additional features to the patient data models, refining the predictive accuracy.

**Figure 1:**
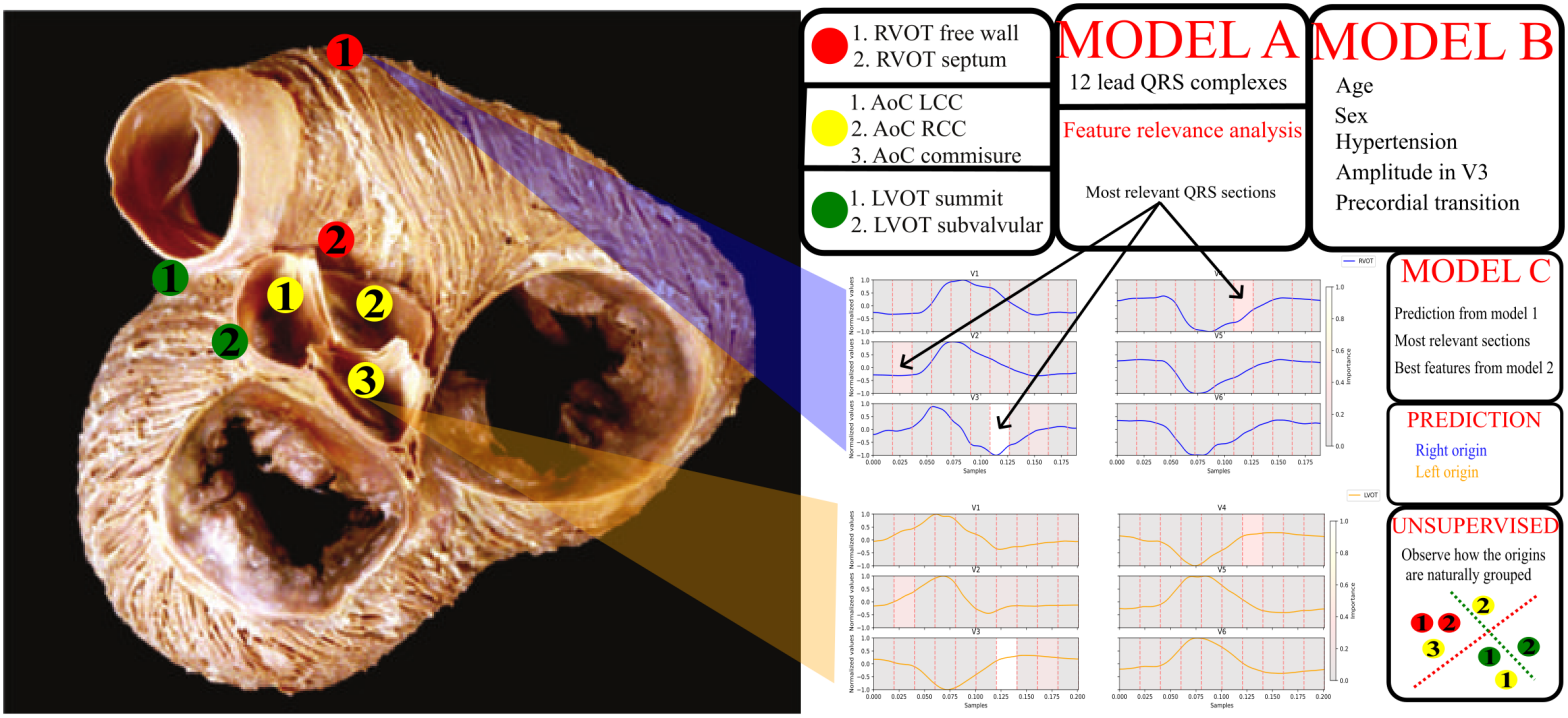
General diagram of the pipeline. On the left, a picture of the outflow tracts adapted from Sánchez-Quintana et al. (13). The specific sites of origin are marked in the picture. The red points correspond to the RVOT origins, the green points to the LVOT, and the yellow to the aortic cusp (AoC) origins, which are subgroup from the LVOT origins. On the right, a legend for the origins, two signal examples for RVOT (blue) and LVOT (orange) origins and a scheme with the models obtained from the different experiments. Model A uses the 12 lead QRS complexes, model B patient data and basic ECG features listed in the figure, model C uses the best QRS segments obtained from the feature relevance analysis of model A, the best feature configuration obtained from model B and the prediction done by the model A. Finally, unsupervised clustering was performed to explore the natural distribution of the specific sites of origin using the best set of features.

Finally, the study employed an unsupervised hierarchical clustering model for exploring the structure within the data. Unlike other models focusing on distinguishing between right (RVOT) and left (LVOT) ventricular origins, the developed model was tailored to identify the specific SOO within the ventricular structure. This unique approach adds depth to the understanding of OTVA and refines the targeting of treatment strategies. The subsequent sections delve into the details of the databases used, the supervised trained models, the analysis of features and models, the unsupervised training methodology for identifying the specific SOO, and the different experiments performed. The overall pipeline is shown in Figure 1.

### 2.1 Databases

We employed four multi-centric ECG databases for our study, each serving a complementary purpose:

• **DS-2496:** Comprising 2469 (RVOT: 1040, LVOT: 1456) simulated 12-lead ECG signals, it was generated to replicate OTVA patients using Doste et al. (14) pipeline.
• **DS-31:** Featuring 31 (RVOT: 17, LVOT: 14) 12-lead ECG cases from Hospital Clínic, Barcelona.
• **DS-334:** Containing an open-source database with 334 RVOT: 234, LVOT: 100) 12-lead ECG signals published by Zheng et al. (15).
• **DS-114:** Consisting 114 (RVOT: 79, LVOT: 35) cases retrieved from Hospital Teknon, Barcelona; these cases were partially used in Penela et al. (9) and included the 12-lead ECG signals, multiple beats from each patient, including the PVC, and clinical data of the patient.

Ethical guidelines were followed, with written informed consent obtained from every patient. To address the lack of standardization in the databases, we developed two distinct models. The first model utilized all four datasets to train models based on QRS complex morphology. The second model leveraged clinical data from DS-114 to train a separate model. Additionally, a third approach combining both models was explored.

The dataset was divided into training (80%) and test (20%) sets using stratified splitting to maintain the proportion of RVOT and LVOT origins (4). Each database was split individually, and the subsets were merged according to the model being trained. A 5-fold cross-validation was performed with a grid search for hyperparameter optimization in both models using the training subset. This meticulous approach ensured robust training and testing procedures for the subsequent analyses.

### 2.2 Supervised models

The model utilizing QRS complexes was trained following the guidelines outlined in Doste et al. (10). For each lead, the QRS complexes were resampled to 10 samples and concatenated into a vector: thus, each sample represented a 10% segment of the QRS complex of a lead. In the morphological analysis, we opted to include all 12 leads instead of solely relying on the most relevant leads reported in previous studies. This decision was made to ensure a comprehensive assessment of the relevance of the QRS complex across all leads.

The model utilizing clinical information was designed based on the weighted hybrid algorithm proposed by Penela et al. (9). In that study, researchers employed age and amplitude in V3, binarized with thresholds of 50 years old and 1 mV. To enhance generalizability and mitigate overfitting, we normalized age dividing it by 100, ensuring its range matched that of the other features. Additionally, we included the raw amplitude, rather than the binarized version. Furthermore, we formulated an algorithm for the automatic determination of the precordial transition. The R/S ratio was calculated for each lead to identify the precordial lead where the R wave began to dominate.

Given that the precordial transition lead is dependent on the cardiac electrical axis in the horizontal plane and patient clinical features (6), we standardized the R/S transition using sinus rhythm information. We calculated the R/S transition for both beats and combined them into a single feature by subtracting both values.

The delineation of the ECG signal for each patient was accomplished using the convolutional neural network designed by Jimenez-Perez et al. (16, 17). Subsequently, cardiac cycles were isolated, extracting the PVC beat and its immediately preceding one. QRS complexes were segmented, and R/S ratios on the precordial leads were calculated for both beats.

A battery of algorithms were tested for both models: the support vector machines (SVM) implementation from scikit-learn (18) called NuSVC, which is a SVM classifier that uses the Nu parameter to control the number of support vectors; multilayer perceptron (MLP); extra trees (ET); random forest (RF) from scikit-learn (18); and XGBoost solution for Python by Chen and Guestrin (19), were tested for both models. The hyperparameters tuned for each model are listed in Table 1.

**Table 1:** Hyperparameters tuned per model.

Model	Parameter	Range	Step
SVM	Nu value	[0.4, 0.6]	0.1
	Kernel	[rbf, linear, poly]	-
	Degree*	[1,5]	1
	Gamma	[scale, auto]	–
	Coef0*	[0,1]	0.5
MLP	Solver	[lbfgs, adam]	–
	Alpha	[1e−4, 1e−5 ,1e−6]	–
	Hidden layers size	[10, 50, 100]	–
	Activation function	[identity, logistic, tanh, relu]	–
RF	Estimators	[100, 500]	100
	Minimum samples to split	[0.1, 1]	0.1
	Minimum samples per leaf	[0.1, 1]	0.1
ET	Estimators	[100, 500]	100
	Minimum samples to split	[0.1, 1]	0.1
	Minimum samples per leaf	[0.1, 1]	0.1
XGBoost	Maximum depth	[1, 10]	1
	Minimum child weight	[1, 6]	0.5
	Gamma	[0, 0.5]	0.1
	Subsample	[0.6, 1]	0.01
	Column sample by tree	[0.6, 1]	0.01
	Regulation alpha	[1e−5, 1e−2, 0.1, 1, 100]	–

* Only for ’poly’ kernel.

The model’s performance was evaluated using two metrics: accuracy and, considering the imbalanced nature of the classes, the macro-average sensitivity. The macro-average sensitivity assigns equal weight to both classes, as shown in Equation 1, ensuring that the contribution made by the LVOT classification (the less frequent category) is not underestimated in the final result:

(1) $$MA=\frac{1}{2}\left(\frac{TP}{TP+FN}+\frac{TN}{TN+FP}\right),$$

being MA the Macro-Average sensitivity, TP the True Positives, TN the True Negatives, FN the False Negatives and FP the False Positives. This can also be interpreted as the weighted sum of the individual sensitivity of each class.

The presented results were obtained using DS-114 as test set. This choice was made to ensure comparability among the results, given that DS-114 is the only database containing both QRS complexes and patient clinical information.

To improve the classification process, we analyzed the feature relevance in the best model of the approach based on QRS complex analysis, using the SHAP tool (11) and Gini’s coefficient (12). The most significant features, identified through this analysis, were retained for their clinical significance. These features were then incorporated into the model based on clinical features. The same methodology was applied to the third approach to identify and retain the most critical features.

### 2.3 Unsupervised models

We aimed to examine how different origins are clustered based on the labels of the specific SOO provided by clinicians. We used hierarchical clustering because of its ability to dynamically organize samples into clusters based on similarity, offering flexibility in accommodating the inherent complexity of ventricular SOOs.

The initial step involved the reduction and consolidation of various labels provided by clinicians. The original 56 labels were condensed into a more manageable set of 7 labels, following the labelling of Penela et al. (9), since some of the cases from DS-114 were used in that study as well. The final labelling includes: right coronary cusp (RCC), left coronary cusp (LCC), RCC/LCC commissure, LVOT sub valvular, LV summit, RVOT septum and RVOT free wall.

Utilizing the meticulously curated dataset, we employed hierarchical clustering to reveal patterns and relationships among distinct ventricular SOOs. We used the Ward’s variance minimization method from scipy to determine how clusters are formed (20, 21), allowing us to group samples without the need to predefine the number of clusters—a crucial advantage in situations where the optimal number of clusters is not predetermined.

### 2.4 Experiments

We devised a comprehensive set of experiments, each providing complementary insights into the challenging task of accurately classifying the SOO as either right ventricular outflow tract (RVOT) or left ventricular outflow tract (LVOT). The experiments focused on supervised approaches, labelled A, B, and C, were designed to harness the power of diverse data sources and methodologies. Experiment D was designed for the unsupervised approach, focusing on exploring different ways to cluster the specific SOO. A summary of the experiments is shown in Table 2.

**Table 2:** Summary of experiments with supervised approaches.

Experiment	Scenario	Input
A	1	QRS complexes from DS-114
	2	QRS complexes from all databases
B	1	BinAge, sex, HTA, BinV3 and PTR
	2	BinAge, sex, HTA, BinV3 and PTC
	3	NormAge, sex, HTA, AmpV3 and PTR
	4	NormAge, sex, HTA, AmpV3 and vPTC
	5	NormAge, sex, HTA, AmpV3 and vPTCps
	6	NormAge, sex, HTA, AmpV3 and vPTCVal
	7	NormAge, sex, HTA, AmpV3 and vPTCpsVal
C	1	Binary prediction of best model in Experiment **A** Best features from best model in Experiment **B**
	2	Probability per class of best model in Experiment **A** Best features from best model in Experiment **B**
	3	Best features from best model in Experiment **A** Best features from best model in Experiment **B**
	4	Binary prediction of best model in Experiment **A** Best features from best model in Experiment **A** Best features from best model in Experiment **B**
	5	Probability per class of best model in Experiment **A** Best features from best model in Experiment **A** Best features from best model in Experiment **B**

BinAge, Binarized age with threshold >50; NormAge, Normalized age; HTA, Hypertension; BinV3, Binarized amplitude in V3 with threshold >1 mV; NormV3, Amplitude in V3; PTR, Precordial transition reported by clinicians; PTC, Precordial transition calculated; vPTC, Precordial transition calculated in vector form; vPTCVal, R/S values of the precordial transition calculated in vector form; vPTCps, Precordial transition calculated considering sinus rhythm and PVC beats in vector form; vPTCpsVal, R/S values of the precordial transition calculated considering sinus rhythm and PVC beats in vector form.

#### 2.4.1 Experiment A: training on QRS morphology

This experiment aimed to train the models exclusively with QRS complexes. Two distinct scenarios were considered. In Scenario 1 (A.1), the model was trained and tested only with dataset DS-114. In the second Scenario (A.2), all other databases were also incorporated, all the test subsets were included in the hyperparameters grid search, but the results were reported using the DS-114 test subset.

#### 2.4.2 Experiment B: training on clinical variables and features from the ECG

First, labels assigned by clinicians were compared with those predicted by our model, considering uncertainties in clinically-defined transitions. Subsequently, the precordial transition vectors were defined for each patient. These vectors, each with a length of six, were created with all elements set to zero, except for the transition point, which was assigned a value of 1. Vectors were built for both calculated and clinically estimated transitions. The dissimilarity between automatic and clinically reported transitions was then assessed using vector shifting, a measure that quantifies positional distance. Diverse models were subsequently trained using both calculated and provided precordial transitions, along with patient-specific clinical information. This comprehensive approach aimed to evaluate the accuracy and alignment between automatically obtained precordial transitions and clinically annotated transitions, considering the broader context of patient-specific clinical data.

A first model was trained with the same information as utilized by Penela et al. (9), serving as the reference (B.1). In the second Scenario (B.2), the calculated precordial lead was employed instead of the clinical annotations. In the third Scenario (B.3), clinical annotations were utilized, but thresholds were removed. In Scenario four (B.4), the calculated PVC transition without thresholds was incorporated. The fifth Scenario (B.5) involved a combined version of the PVC and sinus rhythm transitions without thresholds. In Scenario six (B.6), no thresholds were applied, and the PVC transition utilized the R/S transition rather than the lead name alone. Finally, in the seventh Scenario (B.7), the approach mirrored that of scenario six, but sinus rhythm values were also included.

#### 2.4.3 Experiment C: training on combined QRS morphology and clinical data

In this experiment, we leveraged the optimal model from Experiment A to identify the most relevant QRS features. We conducted inference on the training set and combined these results with the clinical features employed by the best model from Experiment B. Furthermore, the amplitudes identified as the most relevant in Experiment A were integrated. This process resulted in the creation of five new training scenarios. Scenario C.1 included the binary prediction of the best model from Experiment A. Scenario C.2 replaced the binary prediction with the probability per class, accommodating instances where the model’s prediction was less clear. In Scenario C.3, the most relevant amplitudes were introduced as additional features. Finally, Scenarios C.4 and C.5 represent combinations of C.1 and C.2, respectively, with the inclusion of the relevant amplitudes, ensuring a comprehensive exploration of all possible combinations.

#### 2.4.4 Experiment D: unsupervised clustering of the SOO

After identifying the best set of features, we explored the distribution of specific SOO using hierarchical clustering to examine how the origins were dispersed. To determine the optimal threshold at which clusters are not merged, we used grid search while assessing the clustering through the silhouette score.

For better visualization, a heatmap was used to summarize the clustering information. Additionally, the labels were grouped in 3 major structures: aortic cusp (AoC), RVOT and LVOT. Note that although AoC is part of the LVOT, it comprises various distinct labels. Anatomically, these labels are in closer proximity to each other compared to other LVOT structures. Furthermore, due to their proximity to lead V1, they are more likely to manifest a LBBB pattern (6). Interestingly, LBBB patterns are typically associated with an origin in the RVOT. Given the intricate nature of the aortic cusp and its unique characteristics, it justifies a dedicated and focused study.

## 3 Results

### 3.1 Experiment A: training on QRS morphology

**Figure 2:**
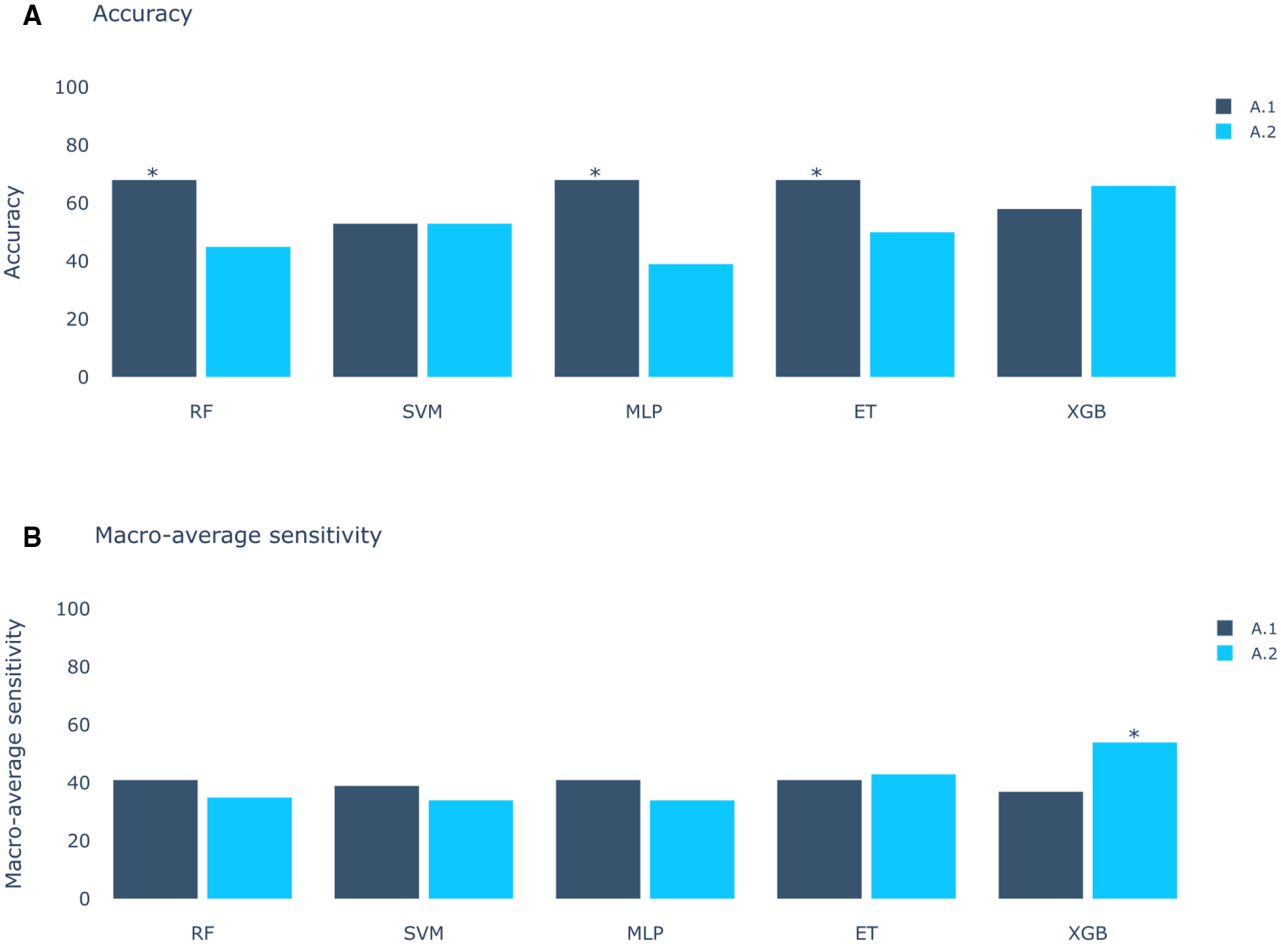
Models score comparison. RF, Random Forest; SVM, Support Vector Machine; MLP, Multilayer Perceptron; ET, Extra Trees; XGB, XGBoost. (**A**) Models accuracy.* shows the highest scores at 68% in RF, MLP and ET for Experiment A, Scenario 1 (A.1). (**B**) Models macro-average sensitivity comparison. * shows the highest score at 54% for XGB in Experiment A, Scenario 2 (A.2). Achieving better results when using all databases (Scenario 2) than using only DS-114 (Scenario 1).

**Figure 3:**
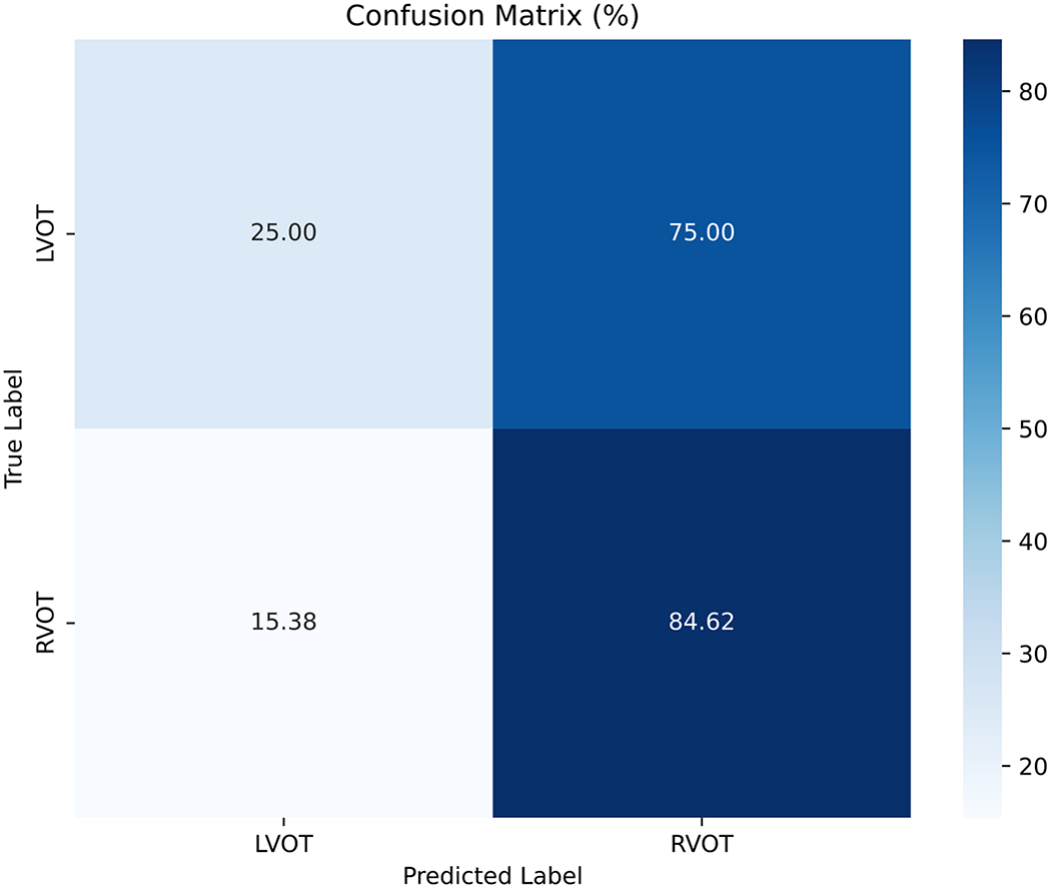
Confusion matrix of the model of Experiment A.2 with XGBoost.

The results of Experiment A (Figure 2) show that Scenario A.1 achieved the highest accuracy in the RF, MLP, and ET models. However, the macro-average sensitivity is comparatively lower. In examining individual class performances, Scenario A.1 demonstrated a 68% accuracy and 41% macro-average sensitivity, aligning with perfect classification for RVOT, but 0% for LVOT. Conversely, in Scenario A.2, the XGB model yielded similar accuracy results (66%) and a higher macro-average sensitivity, indicating that the use of multiple databases for training decreases the risk of overfitting, this can be evidenced in Figure 3 which shows the confusion matrix for the XGB model with a higher performance for LVOT cases.

**Figure 4:**
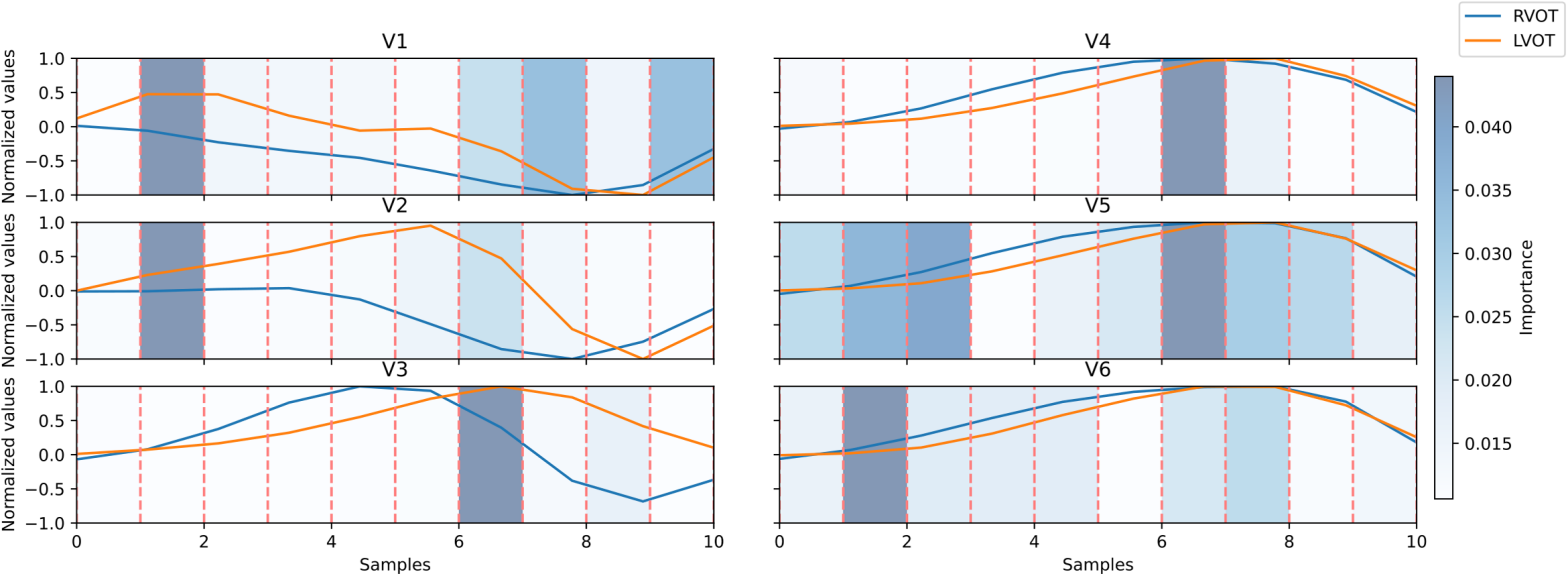
Signal comparison between the average QRS complex of right ventricular outflow tract (RVOT) cases, in blue, vs. left ventricular outflow tract (LVOT) cases, in orange, in the precordial leads. The distribution of relevance per section; in blue scaled in the background, the amplitudes were normalized per lead. Dashed lines in red mark segments of 10% of the original signal.

This XGB model proved to be the optimal in Experiment A. Upon evaluating the Gini’s coefficient of the model, the analysis revealed that the most influential leads were V3, V4, and V2, accounting for approximately 51% of the total cumulative score. This was followed by aVR (9.7%) and V1 (8%). Further examination of the signal indicated that the most critical sections of the QRS complex were V3 between 60%-70% of the cardiac cycle, which corresponds to the transition between the R and the S waves, followed by V4 in the same section of the QRS complex, and V2 in the 10%-20% section, which corresponds to the beginning of the Q wave. The distribution of relevance per section in the most influential leads is depicted in Figure 4.

**Figure 5:**
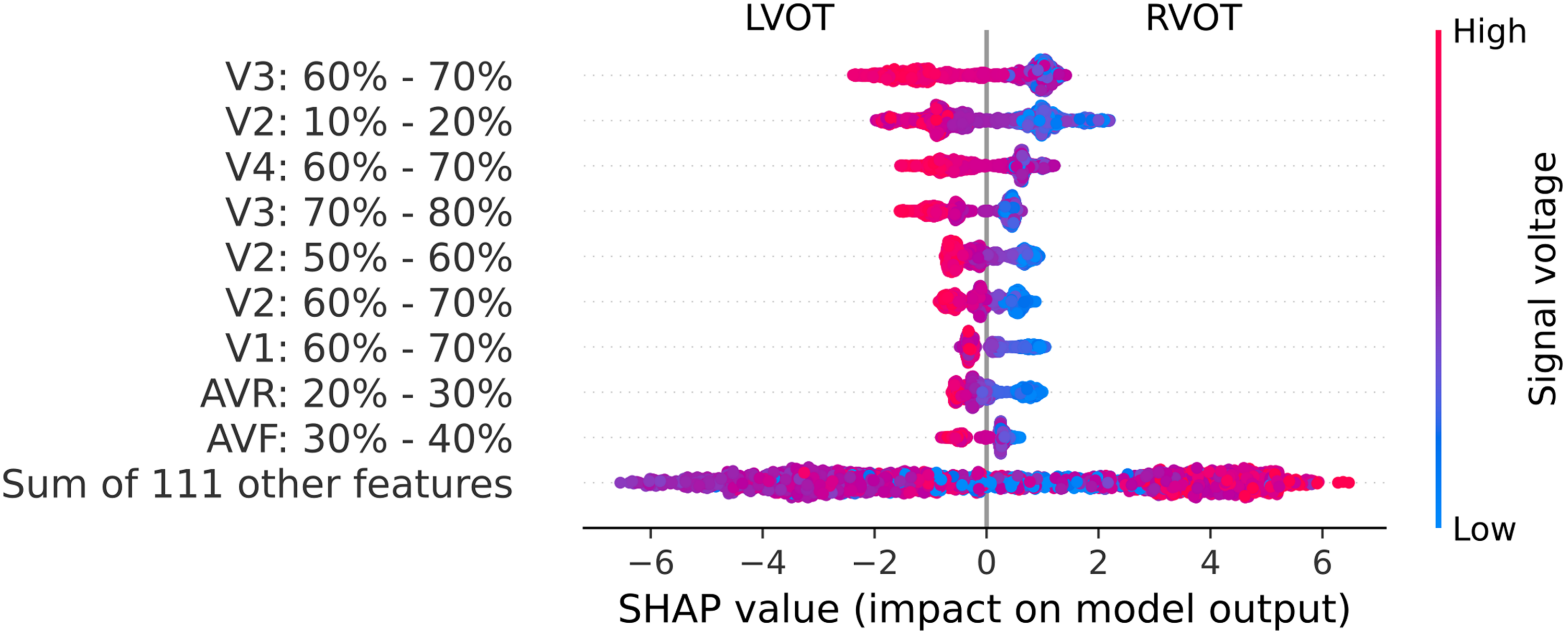
Beeswarm graph from the SHAP values. The SHAP values (horizontal axis) show how each feature (left column) contributes to the negative (LVOT) or positive (RVOT) outputs. Color is employed to represent the original value of a feature, in this case, mean voltage per QRS complex section (each feature corresponds to 10% sections of the QRS complex in each lead). Each dot corresponds to one patient.

These relevant segments identified by the Gini’s coefficient closely align with those identified in the SHAP analysis, as depicted in Figure 5. The beeswarm plot visually represents the salient features and indicates the impact of each feature along the X-axis. The amplitude of the SHAP value reflects the feature’s relevance, with the sign denoting its contribution direction—negative values correspond to LVOT, and positive values to RVOT. Furthermore, warm colors signify higher voltages, while cold colors indicate lower voltages.

Leads V2, V3, and V4 consistently stand out as the most influential, closely followed by V1 and aVR. The beeswarm plot highlights the correlation between low voltage values (blue in Figure 5) in the R/S section of the QRS complexes in V1, V2, V3, and V4 (50%-80% of QRS complex), along with the initial part of the QRS complex in V2, with an association to RVOT origin. Conversely, elevated voltages (orange in Figure 4) in these segments are linked to LVOT. This can equally be observed in Figure 4, in which the differences between LVOT and RVOT origin signals are shown. In the R/S transition segment of V1, V2, V3 and V4, the average normalized voltage of LVOT is higher. The same happens in the initial segment of V2, just before the R wave slope becomes discernible.

### 3.2 Experiment B: training on clinical variables and features from the ECG

When compared with clinician annotations, the automatic ones coincided with the same precordial lead only in 23.5% of instances. However, the average shifting was 1.37, with an interquartile range of 1.5 and a median of 1. This suggests a typical shift of around one lead (e.g., transition between V2 and V3 vs between V3 and V4), with some cases displaying more substantial deviations. The impact of these differences is reflected in the results of the model training.

**Figure 6:**
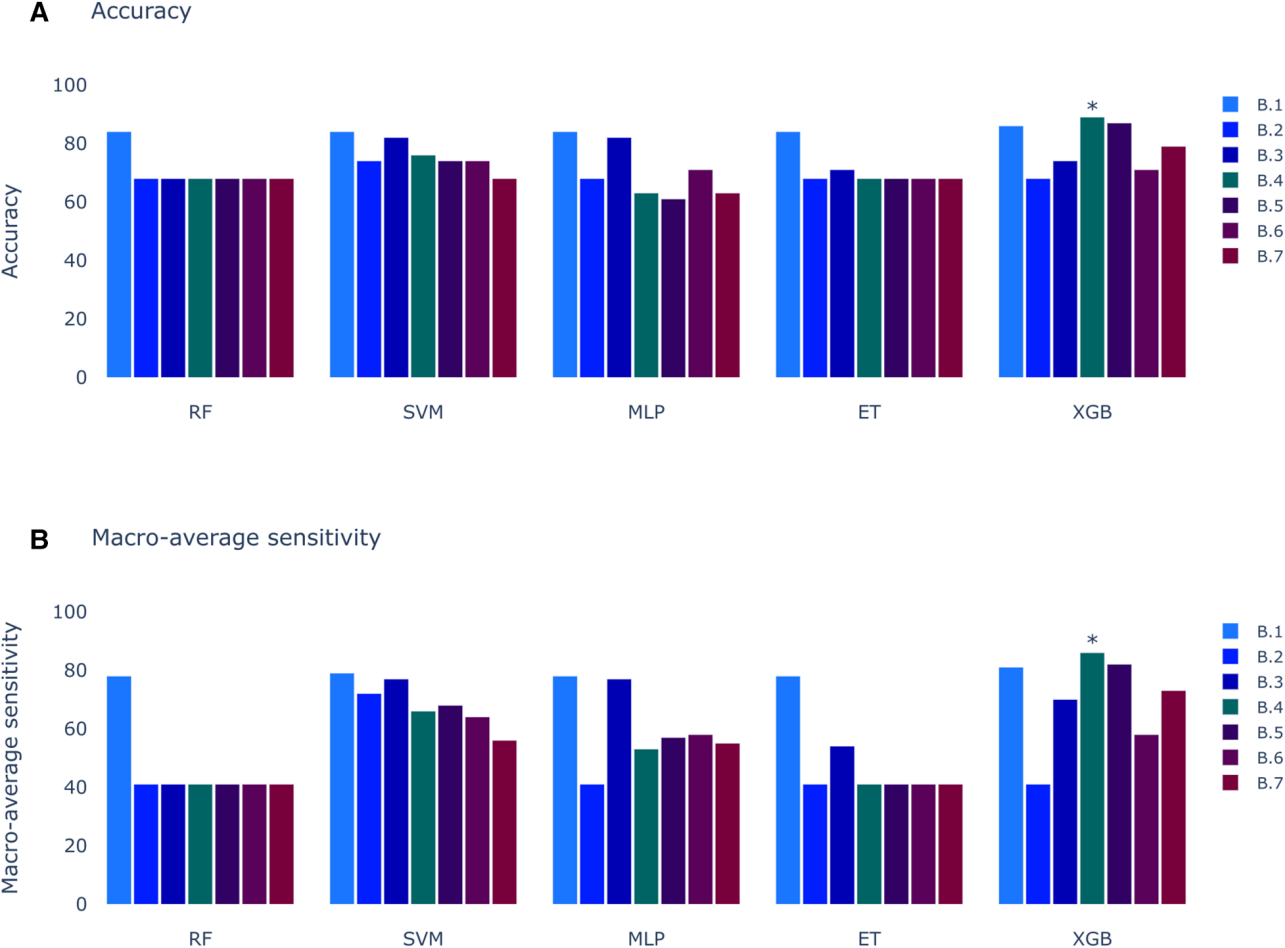
Models score comparison. RF, Random Forest; SVM, Support Vector Machine; MLP, Multilayer Perceptron; ET, Extra Trees; XGB, XGBoost; RF, Random Forest; SVM, Support Vector Machine; MLP, Multilayer Perceptron; ET, Extra Trees; XGB, XGBoost. (**A**) Models accuracy. * shows the highest scores at 89% in XGB for Experiment B, Scenario 4 (B.4). (**B**) Models macro-average sensitivity comparison. * shows the highest score at 86% for XGB in Experiment B, Scenario 4 (B.4).

In contrast to the results presented in Figure 2, the outcomes of the models trained with clinical variables (Figure 6) reveal that the top-performing scenario remains consistent for both accuracy and macro-average sensitivity metrics. Scenario B.4, encompassing automatic precordial transition in vector form, sex, hypertension, amplitude of V3 lead, and normalized age, achieved an impressive 89% accuracy and 86% macro-average sensitivity (see Figure 6). Using Scenario B.1 as a baseline, which only employs the clinical features from Penela et al. (9), we observe a performance decrease with the inclusion of the computed transition (Scenario B.2). Additionally, normalizing age and incorporating V3 lead amplitude (Scenario B.3) did not enhance the performance. Introducing R/S ratio values per lead (Scenarios B.6 and B.7) failed to yield an improvement. Notably, only Scenarios B.4 (89% accuracy and 86% macro-average sensitivity) and B.5 (87% accuracy and 82% macro-average sensitivity) surpassed the scores of B.1 (86% accuracy and 81% macro-average sensitivity). The key distinction between these scenarios is the inclusion of the sinus rhythm transition in B.5. While features from B.1 consistently outperform others across all models, alternatives using the calculated precordial transition for both sinus rhythm and PVC, and PVC alone, outperform Scenario B.1.

### 3.3 Experiment C: training on a combination of QRS morphology and clinical data

**Figure 7:**
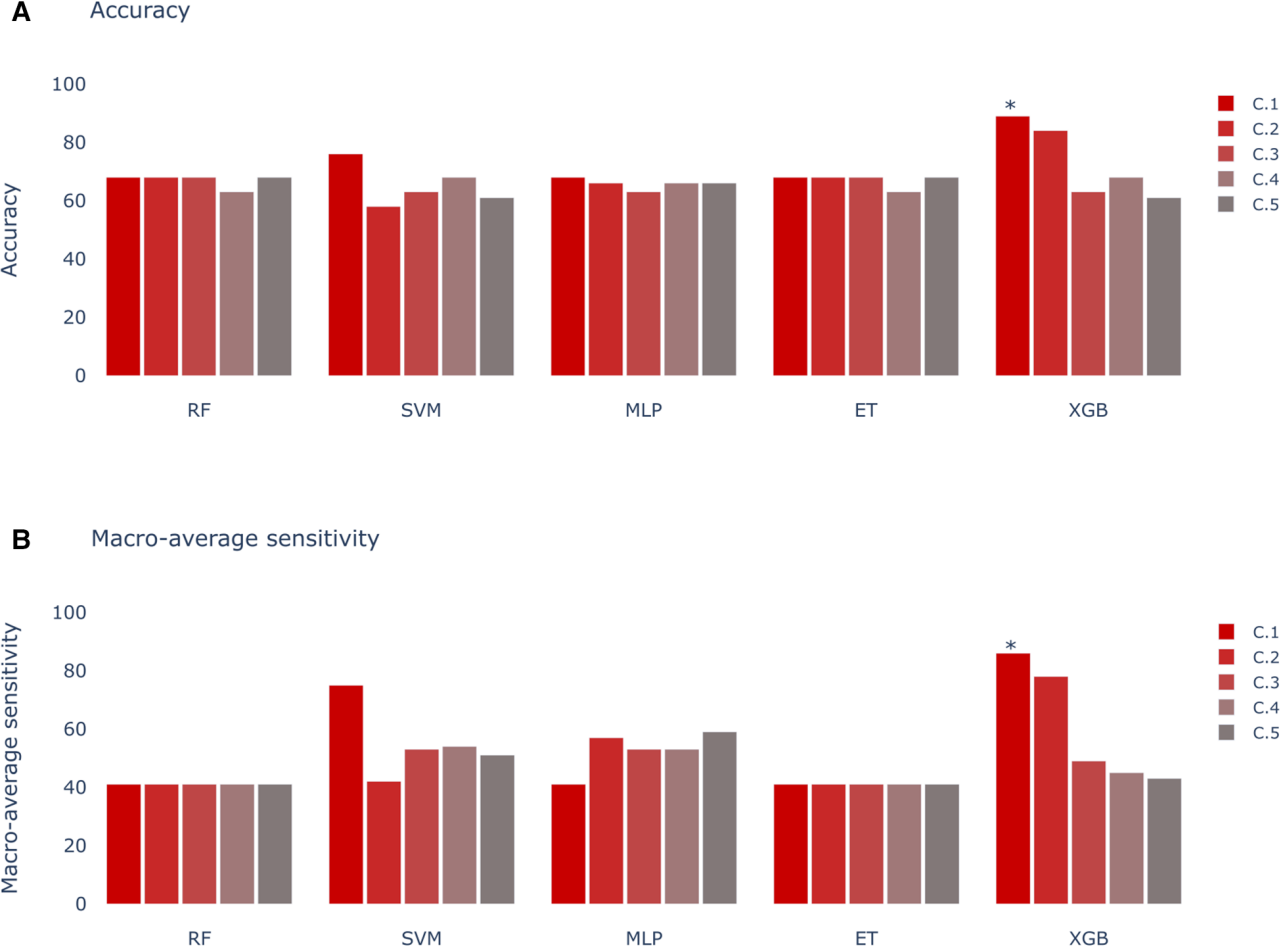
Models score comparison. RF, Random Forest; SVM, Support Vector Machine; MLP, Multilayer Perceptron; ET, Extra Trees; XGB, XGBoost. (**A**) Models accuracy. * shows the highest scores at 89% in XGB for Experiment C, Scenario 1 (C.1). (**B**) Models macro-average sensitivity comparison. * shows the highest score at 86% for XGB in Experiment C, Scenario 1 (C.1).

**Figure 8:**
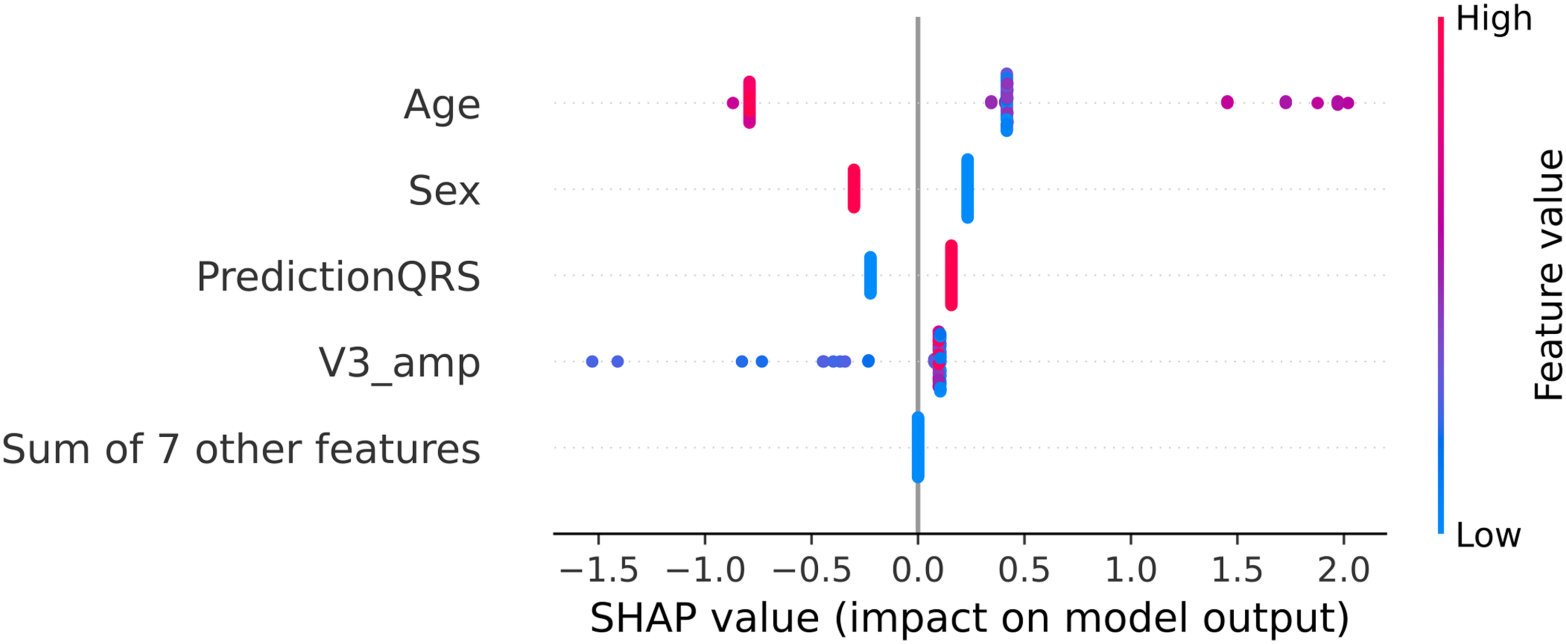
SHAP values for the best model in Experiment C (C.1 with XGBoost). The SHAP values (horizontal axis) show how each feature (left column) contributes to the negative (LVOT) or positive (RVOT) outputs. Color is employed to represent the original value of a feature. Each dot corresponds to one patient. **Age:** age of the patient, red indicates an older patient. **Sex:** sex of the patient, red indicates male patients, **PredictionQRS:** Binary prediction done by the best model of Experiment A.2, red indicates a LVOT prediction. **V3_Amp:** Amplitude in lead V3, red indicates a higher peak voltage in V3.

The outcomes for the third set of experiments are illustrated in Figure 7. Notably, the best results align with the optimal outcomes of the Experiment B, 89% of accuracy and 86% of macro-average sensitivity. Upon scrutinizing the feature relevance of best model in Experiment C (C.1, with the prediction of SOO done by the best model in Experiment A.2 and the features of the best model of Experiment B.4: normalized age, sex, hypertension, amplitude in V3 and calculated precordial transition in vector form) using the Gini’s coefficient, we identified age and amplitude in V3 as the two most crucial features. These were closely followed by the prediction of the best model in Experiment A.2, with sex trailing further behind. Interestingly, this differs from the findings of the SHAP analysis depicted in Figure 8. In this analysis, SHAP values reveal that age and sex emerge as the most relevant features, followed by the prediction of the QRS model and amplitude in V3.

The similarity in results between Experiment C and Experiment B suggests a potential case of redundancy in the features used by the model. Specifically, there appears to be overlap between the prediction generated by the best model in Experiment A.2 and the precordial transition vector. To investigate this further, we conducted a test comparing the performance of a model that excludes both the model prediction from Experiment A.2 and the precordial transition vector, with two other models incorporating each feature separately.

We observed that adding both features separately led to the same increase in model performance, approximately 4.5 percentage points. This finding suggests that both the model prediction from Experiment A.2 and the precordial transition vector may have a comparable impact on the model’s performance.

Furthermore, when comparing the model performance without the precordial transition and model prediction from Experiment A.2 with the model obtained during Experiment C, we noted a decrease in classification performance of 4.5 percentage points. This further supports the notion that both features exert a similar effect on the model.

These findings underscore the importance of considering feature redundancy in model development and suggest potential avenues for optimization in future iterations of the model.

### 3.4 Experiment D: unsupervised clustering of the site of origin

**Figure 9:**
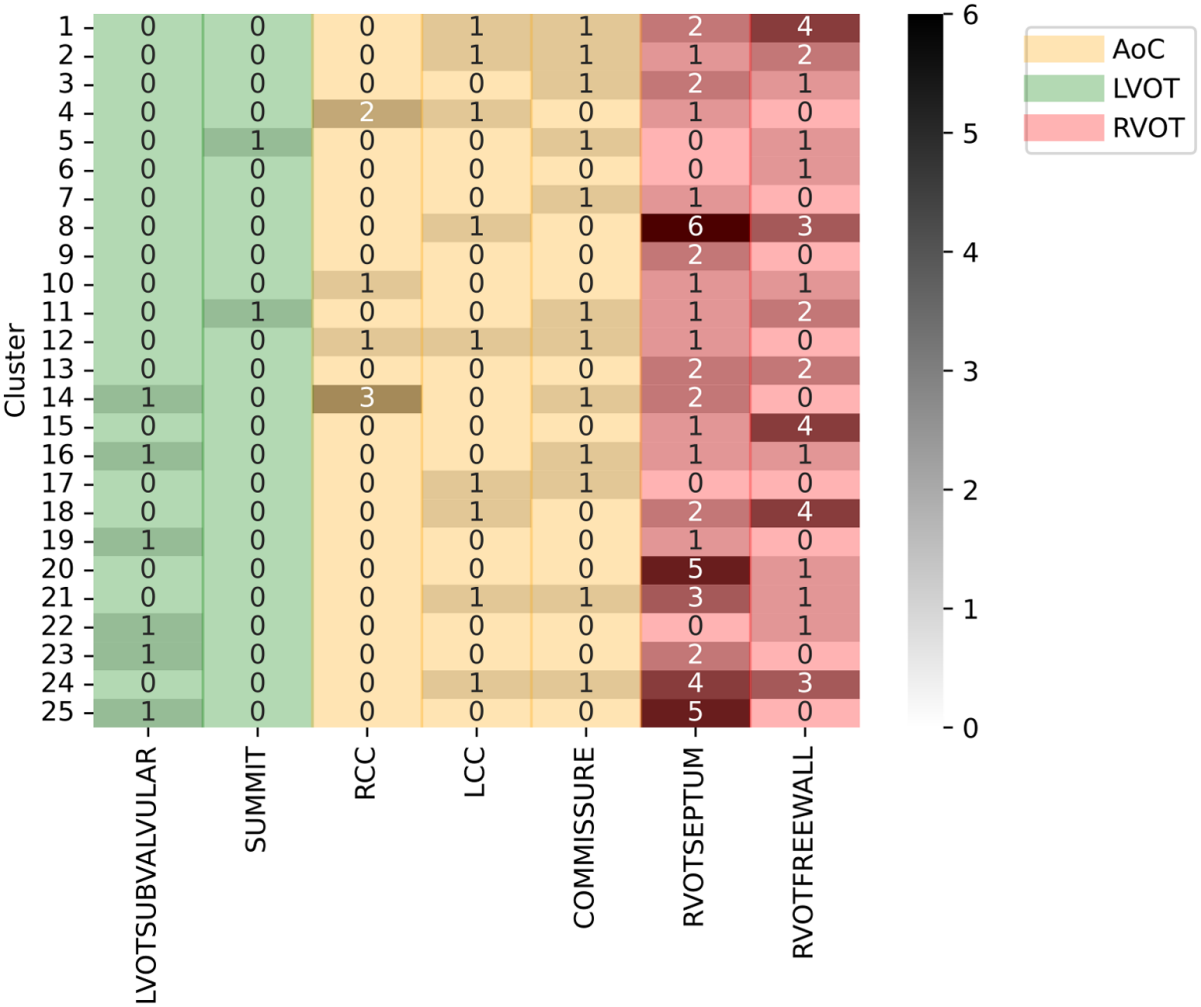
Clustering results using specific SOO as label. Y-axis show the clusters, X-axis show the specific SOO, being: LVOTSUBVALVULAR, left ventricular outflow tract in the sub-valvular area; SUMMIT, summit of the left ventricle; RCC, right coronary cusp; LCC, left coronary cusp; COMMISSURE, RCC/LCC commissure; RVOTSEPTUM, the septum in the right ventricular outflow tract and RVOTFREEWALL, the free wall in the right ventricular outflow tract. Green zone groups the LVOT origins, excluding the AoC origins, the red zone groups the RVOT origins and the yellow zone shows the AoC origins. The grayscale shows the frequency of each origin per cluster.

The silhouette score identified 25 clusters as optimal, thereby shedding light on the inherent data distribution of DS-114. Figure 9 illustrates the clustering results using refined labels, with colors indicating their major region classification (AoC, LVOT, and RVOT). Notably, clusters with similar numbers are closely situated in the dendrogram, implying that the origins in cluster 20, for instance, are closer to those in cluster 25 than to those in cluster 1. To simplify the labels, they were categorized as top (1-8), mid (9-17), and bottom (18-25). In the top category, LVOT cases exhibited a low occurrence, while the majority of RVOT cases originated from the free wall. Within the mid-category, RVOT originating from the septum and free wall were evenly distributed, with an increased presence of aortic cusp cases in clusters featuring more RVOT septum origins. Finally, the bottom section of the heatmap indicates a higher prevalence of RVOT septum cases accompanied by a simultaneous decrease in RCC cases. This suggests that RCC origins tend to cluster more than other AoC origins, and sites associated with the AoC cluster more closely with septal origins. Comparing the density distribution of the RVOT free wall and septum origins, these origins tend to cluster more than others, with notable peak densities in certain clusters (1 and 8 for the free wall and 9 and 21 for the septum).

## 4 Discussion

Radiofrequency ablation procedures have become a prevalent method for treating OTVAs, yet the efficacy of the procedure leaves room for improvement, often marked by a high number of recurrence cases. An integral aspect of refining this process involves meticulous planning, particularly in the identification of the SOO before the procedure commences. Numerous methodologies exist for determining the SOO, encompassing morphological analysis of the ECG, examination of patient clinical data, and signal analysis through ML.

In the context of daily medical practice, the efficiency gains offered by streamlined SOO identification cannot be overstated. By reducing the time required for clinicians to pinpoint the SOO, the proposed approach holds promise for expediting patient care pathways and minimizing procedural risks. Moreover, by integrating data from multiple hospitals and incorporating patient-specific variables such as age and sex, your methodology aims to mitigate biases inherent in existing criteria and deliver more personalized treatment strategies. This holistic approach not only empowers clinicians with actionable insights but also serves as a pivotal step towards standardizing OTVA diagnosis and treatment across diverse patient populations.

The work presented herein introduces a decision system designed to differentiate origins in RVOT and LVOT, building upon the groundwork laid by Penela et al. (9) and Doste et al. (10). Their earlier works involved the utilization of clinical data with visual ECG analysis and multi-center, simulated ECG signals, respectively. In our proposal, three approaches using different input features were designed.

The first approach (Experiment A), inspired by the methodology of Doste et al. (10), exclusively employs QRS complexes. This initial strategy not only facilitated origin classification, but also provided valuable insights into the significance of each segment of the QRS complex, contributing to a deeper understanding of the decision-making process employed by the model. The results of the first approach in Experiment A revealed a peak accuracy of approximately 68%. However, a more comprehensive analysis, including macro-average sensitivity, exposed a significant limitation: the model consistently classified all samples as RVOT origin. This outcome, while yielding a high accuracy percentage owing to the higher prevalence of RVOT origins, poses a risk associated to the inherent class imbalance. In pursuit of a more balanced assessment, the macro-average sensitivity emerged as a pivotal metric, revealing that the most effective result was achieved by the XGBoost model trained with all databases, attaining 61% accuracy and 41% macro-average sensitivity. The importance of macro-average sensitivity is evident when comparing the results of Scenario 1 and 2 in Experiment A. Despite higher accuracy in models like RF, ET, SVM, and MLP, they showed lower macro-average sensitivity compared to the best-performing model. Notably, the top model achieved similar accuracy but higher macro-average sensitivity. Although models from Experiment A.1 aren’t directly applicable, this comparison highlights the significance of macro-average sensitivity.

Although falling short of the 84%–86% range reported by Doste et al. (10), potential discrepancies in preprocessing, particularly in the signal alignment stage, could contribute to this variation in performane. Another important source of discrepancies arises from the differences in the acquisition of various databases, making standardization challenging. Uniformization techniques applied to datasets should enhance the accuracy.

SHAP analysis results revealed that leads V1, V2, V3, and V4 play crucial roles in the R/S transition segment. Elevated voltages in these regions were associated with the origins in the LVOT. This correlation may be attributed to an early R/S transition, where higher voltages signify a less negative peak in the S-wave. According to Anderson et al. (4), an early precordial transition is indicative of LVOT origin. Notably, the early high amplitude observed in V2 may be linked to the ascending slope of the R-wave, as depicted in Figure 4. This specific aspect of the QRS complex highlights the differences in the R-wave between the LVOT and RVOT origins.

The second approach (Experiment B) is focused on a combination of clinical variables and some features from the ECG, as proposed by Penela et al. (9). To enhance generalization and mitigate overfitting, this approach eliminated thresholds and labels that were dependent on clinician criteria. In addition, it introduced an innovative algorithm for determining the precordial transition based on the R/S amplitude in the precordial leads, assigning a unique value to each lead that encapsulates the transition point. Optimal performance (accuracy 89%, macro-average sensitivity 86%) was observed when binarizing these values and converting them into a zero vector, with one at the calculated transition point (precordial transition calculated in vector form; see Table 2 for other forms to report the precordial transition). When comparing these algorithm-generated transitions with those determined by clinicians, alignment was achieved in only 23.5% of the cases. However, an in-depth analysis of the discrepancies revealed notable consistency: the majority of differences were confined to a single lead, often with atypical values. These deviations may be attributed to the variability in signal selection. The uncertainty about whether the signals retrieved from the number of available ECG accurately corresponded to those employed by clinicians for the initial diagnosis introduces another potential source of discrepancy. This underscores the importance of signal consistency when aligning algorithmic results with clinical annotations.

Although our accuracy results fall below those obtained by Penela et al. (9) (89% vs. 94%), their approach involved manually setting thresholds and selecting precordial transitions, introducing subjectivity into the process. In contrast, our approach eliminates dependence on thresholds or manual decision systems, making it more resilient to variations in clinical judgments and databases from different hospitals.

The third approach (Experiment C) introduced a novel strategy by leveraging the inference of the first model along with the most influential features identified in the best model of the second approach. This iterative process is crucial in refining the combination of the QRS complex and clinical data, leading to a comprehensive and robust decision system.

Metrics obtained in models for Experiment C did not surpass those obtained in Experiment B, indicating that the addition of raw QRS complexes to the clinical data and most relevant ECG features did not significantly enhance the overall performance of the system. This finding underscores the complexity inherent in the XGBoost model, particularly in the tuning of hyperparameters, which may inadvertently increase the complexity of individual trees. Consequently, the impact of introducing new features from Experiment A may be attenuated by the existing relationships captured by the features in Experiment B. Furthermore, upon analysis of the SHAP values in Figure 8, the prediction of the model from Experiment A is consistently ranked among the top 3 features, underscoring its influence on the decisions of the model from Experiment C. Despite the comparable performance of Models B and C, it is essential to acknowledge that they may employ distinct intrinsic pathways to achieve their respective results.

Alternatively, the precordial transition and the amplitude in V3 may be enough to capture the main differences in the ECG between LVOT and RVOT origins. Nevertheless, feature analysis provided valuable insights. Age, sex, and the binary prediction of the best model in Experiment A.2 emerged as the most crucial features, followed by the amplitude in V3. Interestingly, the precordial transition vector has diminished importance in this approach, possibly due to redundancy with the best model in Experiment A.2. This was tested through an experiment in which the performance of the model was compared by removing both features, revealing that the impact on the final model is similar. Similarly, the most relevant amplitudes contributed to the decrease in the performance of the model.

When comparing these results with the weighted hybrid score designed by Penela et al. (9), the expected behavior was similar: older male patients with a high voltage amplitude in V3 were more likely to have an LVOT origin. However, the SHAP analysis in Figure 8 indicates that the impact of the precordial transition is minimal. Additionally, the Gini coefficient showed that age and amplitude in V3 were the most relevant features. When comparing the SHAP and Gini rankings, the top features remain unchanged; however, the order is different. This could be because of the number of samples used for each method; while the Gini coefficient is based on the samples used to train the system, SHAP uses the complete dataset. Overall, the performance of this approach is comparable with other solutions (9, 10), with the addition of clinical data and the elimination of thresholds and visual analysis, which powers its generalization capabilities.

An unsupervised approach was chosen for the analysis of specific SOOs. This decision was made because of the challenges associated with directly addressing this problem through a supervised approach. The limited number of cases for certain source sites, such as the summit in LVOT (only two cases), and the uneven distribution of cases among classes, could lead to challenges in hyperparameter optimization, potentially resulting in overfitting. Instead, our approach aims to understand how data are naturally distributed. By employing an unsupervised methodology, we sought to identify the sites that clustered together most effectively. This analysis helps us discern the optimal strategy for decomposing the problem and developing a specific SOO classification system that performs as effectively as binary classification.

The results of the unsupervised analyses suggest that sites associated with the AoC tend to cluster more closely with septum origins. Moreover, there is a tendency for RCC origins to cluster more consistently than LCC and LCC/RCC commissure origins, which exhibit a more scattered distribution across clusters. This clustering pattern might be influenced by certain RCC origins being closer to the posterior region of the RVOT than to the septum or summit.

Furthermore, there is a clear distinction between origins from free wall and septum, often being grouped in some clusters, which suggest that these origins are easier to classify. While LVOT origins, excluding AoC, do not display a clear pattern due to their limited occurrences, this preliminary exploration of specific SOO classifications provides valuable insights, suggesting a tiered classification approach, starting with the identification of AoC and RVOT and then proceeding accordingly.

While the developed system shows promise, it is not without limitations. Firstly, the preprocessing applied to the model using QRS complexes results in the elimination of high-frequency features within the signal. This limitation hampers the identification of certain patterns, both in the model itself and in subsequent analyses. Additionally, there is a substantial imbalance in the database, with a lower number of LVOT cases. While signal simulations partially mitigate this issue, including clinical information from patients remains a challenge for the model. This imbalance is also evident in the unsupervised analysis of specific SOO. Future efforts should focus on incorporating tools capable of handling signals with higher frequencies and implementing strategies to address the scarcity of LVOT data.

## 5 Conclusions

The proposed method has demonstrated its effectiveness in classifying LVOT and RVOT origins. By incorporating signal analysis and clinical data, the approach eliminates thresholds and manual analyses, reducing the potential for inconsistencies in diagnosis while maintaining interpretability. However, to further enhance the system, additional analyses with a broader set of features are recommended. Collecting more cases is also essential for improving the system’s generalization power. Preliminary insights into identifying the specific SOO suggest the presence of discernible patterns. Future developments could capitalize on these patterns to create a robust system capable of accurately identifying specific SOOs with varying levels of precision.

## Data availability statement

The raw data supporting the conclusions of this article will be made available by the authors, without undue reservation.

## Ethics statement

The studies involving humans were approved by Instituto del corazón, Centro Médico Teknon. The studies were conducted in accordance with the local legislation and institutional requirements. Written informed consent for participation was not required from the participants or the participants’ legal guardians/next of kin in accordance with the national legislation and institutional requirements.
